# Artificial intelligence for opportunistic screening of osteoporosis across multiple imaging modalities: a systematic review

**DOI:** 10.3389/fmed.2026.1870486

**Published:** 2026-07-31

**Authors:** Xiang Chen, Wanling Wu, Jianying Shen, Yuyan Chen

**Affiliations:** Yuhuan Second People’s Hospital, Taizhou, Zhejiang, China

**Keywords:** artificial intelligence, fracture risk prediction, opportunistic screening, osteopenia, osteoporosis, systematic review

## Abstract

**Background:**

Osteoporosis often remains undetected until fracture. Dual-energy X-ray absorptiometry (DXA) screening is limited by poor accessibility. Opportunistic screening of routine medical images, powered by artificial intelligence (AI), may enable automated, large-scale bone health assessment.

**Methods:**

This systematic review followed PRISMA 2020 guidelines. We searched PubMed, Cochrane Library, and Web of Science up to February 2026 for studies developing AI models for opportunistic osteoporosis screening. Two reviewers independently screened, extracted data, and assessed risk of bias using phase classification and PROBAST. Outcomes included osteoporosis, osteopenia, and fracture risk prediction, evaluated mainly by area under the curve (AUC). Principal component analysis (PCA) was used to explore sources of heterogeneity.

**Results:**

Of 57 included studies (2019–2026), most were retrospective and single-center. Computed tomography (CT) was the most common modality (38 studies), and the spine was the most frequent ROI. Deep learning (29 studies) has largely replaced traditional machine learning, and foundation models have recently emerged. Model performance was generally high (AUC range 0.630–1.000 across tasks), but heterogeneity was substantial. PCA identified sample size, number of centers, and single-center design as major drivers of heterogeneity, smaller and single-center studies tended to report higher AUCs, suggesting possible overestimation. PROBAST assessment revealed that the absence of external validation was the leading source of bias, only 36% of studies conducted external validation, with additional concerns in participant selection and analysis domains.

**Conclusion:**

AI-based opportunistic osteoporosis screening has progressed rapidly with encouraging performance. However, the evidence base remains dominated by retrospective single-center studies with limited external validation, which hinders clinical translation. Future work should prioritize multicenter collaboration, prospective validation, and standardized reporting.

**Systematic review registration:**

https://www.crd.york.ac.uk/PROSPERO/view/CRD420261451716, identifier PROSPERO (CRD420261451716).

## 1 Introduction

Osteoporosis is one of the most prevalent skeletal diseases worldwide (1, 2). It is characterized by low bone mass and deterioration of bone microarchitecture, which increase bone fragility and susceptibility to fracture. In the United States, osteoporosis affects ~26% of women aged ≥65 years and more than 50% of those aged ≥85 years, with an even higher prevalence among postmenopausal white women (3). Globally, nearly 1/3 of women and 1/5 of men older than 50 years will experience an osteoporotic fracture during their lifetime (4). Fragility fractures, particularly of the hip and vertebrae, are the main clinical consequence and are associated with substantial morbidity, increased mortality, and considerable healthcare costs (5–7).

Osteoporosis often remains undetected until a fracture occurs (8). Although diagnostic criteria are well-established, underdiagnosis and undertreatment remain common in clinical practice. At present, diagnosis mainly relies on bone mineral density (BMD) measured by dual-energy X-ray absorptiometry (DXA), with a T-score ≤ − 2.5 indicating osteoporosis (9). However, limited access and imperfect sensitivity reduce its value as a population screening tool (10). A meta-analysis of 19 studies showed that quantitative computed tomography (QCT) identified substantially more osteoporosis cases than DXA (11). Screening rates in routine care also remain low. Among patients with upper-extremity fragility fractures, only 51% underwent DXA, 25% were referred for endocrine evaluation, and 20% initiated pharmacological treatment, with markedly lower DXA utilization in men than women (18% vs. 55%) (12). Screening gaps are also evident in younger individuals. In a fracture liaison service cohort of 1,248 patients, 61.9% were younger than 65 years, yet only 34% met the International Society for Clinical Densitometry (ISCD) criteria for BMD testing (13). These findings suggest that current screening strategies may fail to capture a substantial proportion of at-risk individuals.

To address these limitations, opportunistic screening has gained increasing attention (13, 14). This strategy uses imaging obtained for other clinical purposes to evaluate bone health during routine care, without additional radiation exposure or extra patient visits. Its value is supported by the widespread use of computed tomography (CT), especially in older adults, who frequently undergo chest, abdominal, or pelvic CT scans for unrelated indications. Reanalyzing these existing scans can help identify patients at high risk of osteoporosis with minimal additional burden. CT remains the most extensively studied modality for opportunistic screening. Since a landmark study in 2013 established its feasibility (15), subsequent research has confirmed its diagnostic performance (16–19). More recently, advances in image analysis have extended opportunistic screening to conventional radiographs and magnetic resonance imaging (MRI), thereby further broadening its clinical applicability (20).

In parallel, artificial intelligence (AI), particularly deep learning, has rapidly advanced this field (21, 22). Although clinical adoption remains limited, AI offers important advantages by automating key steps in the screening workflow, including region-of-interest (ROI) selection, image segmentation, BMD estimation, and risk prediction. This may improve efficiency, consistency, and reproducibility. More recently, task-agnostic foundation models, which have become standard in natural language processing and computer vision, have begun to attract attention in medical image analysis. Transfer learning from such models has proven effective for segmentation and classification tasks (23), and models such as CheXagent and DINOv2 are now being explored for chest radiograph interpretation. In osteoporosis diagnosis, applications remain scarce but are emerging. For instance, a recent study compared natural-domain and medical-domain foundation models for opportunistic osteoporosis screening and found that, after appropriate fine-tuning, the medical-domain models did not outperform their natural-domain counterparts (24). This finding challenges the common assumption that domain-specific pretraining is always necessary, and suggests that fine-tuned natural-domain foundation models may suffice for certain medical imaging tasks, potentially lowering data and computation barriers. Despite this promise, the role of foundation models in opportunistic osteoporosis screening has not been systematically reviewed.

Previous reviews have highlighted the promise of AI-based CT screening. One review, which searched the literature up to December 2023, identified 14 studies and reported high accuracy for BMD prediction and osteoporosis classification. However, its relatively early search end date means that a substantial number of more recent publications were not captured. A more recent review included 55 studies, but its scope was broader and not limited to opportunistic screening (25, 26). Importantly, both reviews focused exclusively on CT, whereas our review additionally synthesizes evidence from radiography and MRI, including DWI and ADC MRI, which have shown promising predictive performance in the past 2 years (27, 28).

This review examines the use of AI for opportunistic osteoporosis screening across multiple imaging modalities. We summarize the characteristics of existing studies, evaluate their methodological quality, explore major sources of heterogeneity, and discuss barriers to clinical implementation and future research priorities. By providing a comprehensive overview of the current evidence, this review aims to clarify the status of the field and highlight the clinical potential of AI-driven opportunistic screening.

## 2 Materials and methods

This systematic review was conducted in accordance with the Preferred Reporting Items for Systematic Reviews and Meta-Analyses (PRISMA 2020) guidelines. The complete PRISMA checklist is provided in Supplementary Table 1.

### 2.1 Search strategy

A comprehensive literature search was performed in PubMed, Cochrane Library, and Web of Science from inception to February 28, 2026. The search strategy was developed by the research team and combined Medical Subject Headings (MeSH) terms with free-text keywords. The full search strategy is detailed in Supplementary Table 2.

### 2.2 Eligibility criteria (PICOS framework)

The eligibility criteria were established according to the PICOS (Population, Index test, Comparator, Outcomes, and Study design) framework. The population comprised individuals undergoing routine medical imaging examinations for whom opportunistic osteoporosis screening was applicable. The index test consisted of artificial intelligence-based algorithms applied to medical images. The comparator included DXA or QCT as the reference standard. The primary outcome of interest was the area under the receiver operating characteristic curve (AUC), which served as the quantitative metric for diagnostic performance. Eligible studies included retrospective or prospective studies reporting the development of AI models for opportunistic osteoporosis screening.

### 2.3 Study selection

Title and abstract screening were conducted independently by two reviewers (Xiang Chen and Wanling Wu) according to predefined exclusion criteria. Records were excluded if they were reviews, editorials, conference abstracts, studies unrelated to model development, studies not focused on osteoporosis screening, studies using non-standard imaging modalities, or non-English publications. Inter-reviewer agreement was assessed using Cohen’s Kappa, calculated with the “kappa2” function from the R package “irr” (v0.84). The agreement for this phase was good (Kappa = 0.81, 95% confidence interval (CI): 0.73–0.89).

For full-text screening, the complete texts of 134 articles were retrieved and independently assessed by the same two reviewers (Xiang Chen and Wanling Wu). Further exclusions were made for the following reasons: non-opportunistic screening, absence of model development, missing model performance metrics, unavailability of the full text, or inconsistent reporting of participant numbers. Inter-reviewer agreement was again evaluated using Cohen’s Kappa, yielding a value of 0.86 (95% CI: 0.77–0.95), indicating very good agreement. All disagreements were resolved through discussion or, when necessary, by consultation with a third reviewer (Yuyan Chen). The remaining studies were included in the final analysis.

### 2.4 Data extraction

A standardized data extraction form was developed prior to data collection to capture information on data source, sample size, study population, study design, imaging modality, research question, image segmentation approach, clinical and imaging features, validation method, reference standard, model type, etc. Two reviewers (Xiang Chen and Wanling Wu) independently extracted data from each included study. Any disagreements were resolved through discussion, with a third reviewer (Yuyan Chen) consulted when consensus could not be reached. The primary outcomes of interest were the prediction of osteoporosis, osteopenia, or fracture risk.

From each included study, the area under the curve (AUC) was extracted as the primary metric of model performance. When multiple models or AUC values were reported in a single study, only one AUC was selected according to the following pre-specified hierarchical criteria (applied in order): (1) The AUC of the primary or main model explicitly designated by the authors (e.g., described as the “primary model,” “final model,” or “best-performing model”); (2) The AUC from external validation (independent test cohort), prioritized over internal validation or development set results. If both internal and external validation results were reported, the external validation AUC was extracted even if it was lower than the internal AUC; (3) For studies reporting multiple external validations, the AUC from the validation cohort most similar to our target setting, or the pooled result across external sets if presented by the authors; (4) As a last resort, when none of the above criteria could be applied, the highest reported AUC.

This hierarchical approach was designed to balance methodological rigor with the need for consistent data extraction across all 57 included studies. We acknowledge that selecting the highest AUC in certain cases may still risk some overestimation of performance. This limitation is discussed further in the Limitations section.

### 2.5 Quality assessment

The methodological quality of all included studies was independently assessed by two reviewers (Xiang Chen and Wanling Wu), with cross-checking to ensure consistency. Inter-reviewer agreement was quantified using Cohen’s Kappa for binary classifications, computed with the “kappa2” function from the R package “irr” (v0.84). All disagreements were resolved through discussion or, when necessary, by consultation with a third reviewer (Yuyan Chen).

#### 2.5.1 Model quality assessment

Model quality was evaluated using the Phase classification criteria, which consider sample size (<100 or ≥100), study design (retrospective or prospective), validation approach (internal or independent/external), and development phase (pre-market or post-market). According to this framework, imaging-based studies are typically classified as Phase II or III. Inter-reviewer agreement was 98.25%, with a Kappa of 0.66.

#### 2.5.2 Risk of bias assessment

The risk of bias and applicability of the included studies were assessed using the Prediction model Risk Of Bias Assessment Tool (PROBAST). This tool evaluates four domains: participants, predictors, outcome, and analysis, and provides an overall judgment of risk of bias. Each domain contains 2, 3, 6, and 9 signaling questions, respectively. Responses are categorized as “yes/probably yes,” “no/probably no,” or “no information.” A domain was rated as high risk of bias if at least one signaling question was answered “no” or “probably no.” The overall risk of bias was considered low only if all four domains were judged to be at low risk. Inter-reviewer agreement for the overall ROB judgment was good, with a Cohen’s Kappa of 0.75 (95% CI: 0.58–0.92, observed agreement = 87.72%).

### 2.6 Principal component analysis (PCA)

To investigate potential sources of heterogeneity, study-level principal component analysis (PCA) was performed separately for studies predicting osteoporosis and osteopenia. Thirteen study characteristics were included: three numerical variables (AUC, number of participants, and number of centers) and ten categorical variables (prediction outcome, reference standard, external validation status, multicenter status, country, study design, gender, region of interest category, imaging modality, and feature extraction method). Numerical variables were standardized, and categorical variables were one-hot encoded. Each study was treated as an independent observation. PCA was conducted using the “prcomp” function in the R package stats (version 3.6.2), and the results were visualized with the “fviz_pca_biplot” function in the R package “factoextra” (version 2.0.0).

## 3 Results

### 3.1 Literature search and study selection

A comprehensive literature search was conducted in PubMed, Cochrane, and Web of Science (last updated February 28, 2026). The detailed search strategy and key terms were described in the Methods section and Supplementary Table 2. In addition, the reference lists of relevant articles were manually screened to identify potentially eligible studies.

A total of 383 records were initially identified (154 from PubMed, 8 from Cochrane Library, and 221 from Web of Science). After removal of duplicates, 265 unique records remained and were subjected to title and abstract screening. Of these, 133 records were excluded for the following reasons: conference abstracts (*n* = 29), reviews or editorials (*n* = 45), non-English publications (*n* = 1), non-routine imaging modalities (*n* = 1), absence of artificial intelligence model development (*n* = 35), and irrelevance to osteoporosis screening (*n* = 22). The remaining 132 articles, supplemented by 2 additional studies identified during the peer review process, brought the total to 134 articles that proceeded to full-text assessment.

During full-text review, 77 articles were excluded due to: non-opportunistic screening design (*n* = 21), inconsistency between reported participant numbers and model development cohorts (*n* = 1) (29), focus on applying existing AI tools rather than developing new models (*n* = 38), lack of clear performance metrics (*n* = 16), and unavailability of the full text (*n* = 1). Finally, 57 studies met the inclusion criteria and were included in the systematic review.

**Figure 1:**
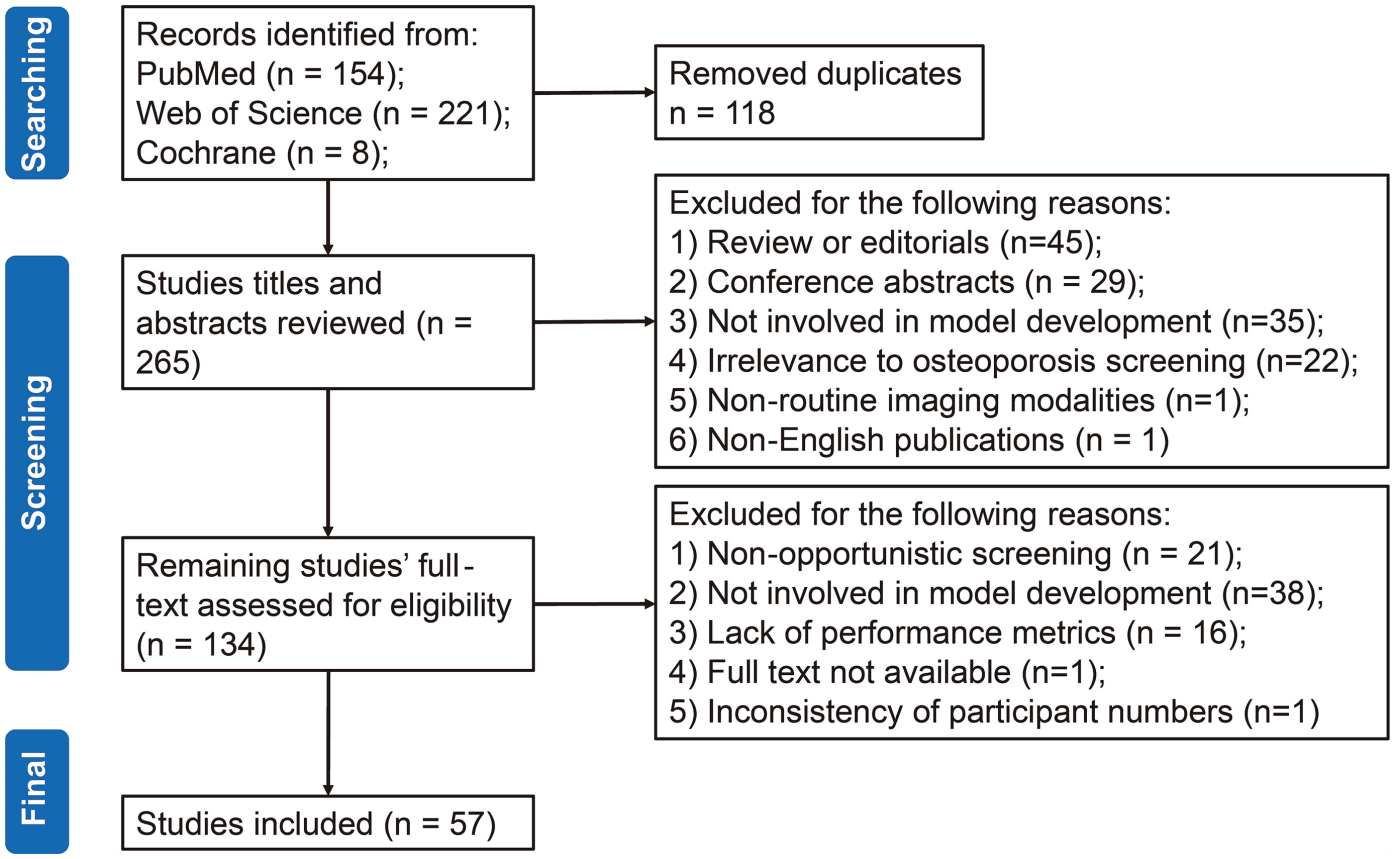
PRISMA flowchart of included studies.

Study selection was performed independently by two reviewers, and discrepancies were resolved through discussion and consensus, resulting in a high level of inter-reviewer agreement. The study selection process is illustrated in the PRISMA flow diagram (Figure 1).

### 3.2 Study and population characteristics: rapid growth with substantial heterogeneity

#### 3.2.1 Study design characteristics

A total of 57 studies were included. Detailed information is provided in Table 1. Publication years ranged from 2019 to 2026. The majority of studies (*n* = 45, ~78%) were published from 2023 onward (30–72), with a particularly high concentration in 2025 and early 2026 (*n* = 19), indicating that AI-based opportunistic osteoporosis screening has entered a phase of rapid expansion. Geographically, the studies were dominated by mainland China, which contributed approximately 47% of all studies (*n* = 26) (34–36, 39–42, 44, 45, 47, 48, 51, 52, 55, 57, 59–62, 64, 67, 69, 71, 73–75). Other contributing countries included the United States (*n* = 7) (46, 65, 68, 76–79), South Korea (*n* = 6) (38, 50, 53, 54, 80, 81), and Italy (*n* = 3) (31, 43, 82), with smaller numbers of studies originating from Taiwan (33, 56, 58, 66, 83), Finland (84), Germany (85), Japan (32, 63), Spain (30), Switzerland (49), Turkey (72, 86), as well as two multi-region studies (37, 70). This geographic imbalance may reflect regional differences in imaging utilization and varying levels of institutional and policy support for AI adoption.

**Table 1:** Characteristics of study design.

ID	First author	Publication year	Participant	Training	Validation	Testing	Cross-validation	External validation	Multi-center	Number of centers	Country	Study design
1	Wei L.	2026	1,306	70%	30%	NA	No	No	No	1	China	Retrospective
2	Birtolo M. F.	2026	109	65	44	NA	No	No	No	1	Italy	Retrospective
3	Hiyama A.	2026	123	98	25	NA	Yes	No	No	1	Japan	Retrospective
4	Ho U-C.	2026	218	80%*	20%*	NA	Yes	No	No	1	Taiwan, China	Retrospective
5	Acosta-Batlle J.	2025	1893	77%*	23%*	313*	No	No	No	1	Spain	Retrospective
6	Jiyoung Song	2026	383	268	115	NA	Yes	No	No	1	South Korea	Retrospective
7	Li Y.	2025	4,305	1891	806	1,608	No	Yes	Yes	5	China	Retrospective
8	Cheng Jia	2025	161	113	48	NA	Yes	No	No	1	China	Retrospective
9	Li Y.	2025	1,167	495	169	503	No	Yes	Yes	4	China	Retrospective
10	Park J.	2025	75,533	46,048	6,498	22,987	No	Yes	Yes	>5	South Korea, United States, Latin America, etc.	Retrospective
11	Tang J.	2025	1780	1,184	296	300	Yes	No	No	1	China	Retrospective
12	Ren Q.	2025	509	279	120	110	No	Yes	Yes	2	China	Retrospective
13	Li Y.	2025	987	561	177	249	No	Yes	Yes	4	China	Prospective
14	Fanelli F.	2025	301	241	60	56	No	Yes	Yes	2	Italy	Retrospective
15	Du C.	2025	456	248	107	101	No	No	No	1	China	Retrospective
16	Wu Y.	2024	3,312	2,337	975	4401*	No	Yes	No	1	China	Retrospective
17	Farid Gharehmohammadi	2025	907	Unspecified, total 729		178	Yes	No	No	1	United States	Retrospective
18	Chengbin Huang	2025	1,126	581	NA	545	No	Yes	Yes	4	China	Retrospective
19	Qiye Cheng	2024	307	214	93	NA	No	No	No	1	China	Retrospective
20	Fabio Galbusera	2024	429	Unspecified, total 373		86	Yes	No	No	1	Switzerland	Retrospective
21	Heejun Park	2024	420	Unspecified	Unspecified	Unspecified	No	Yes	Yes	2	South Korea	Retrospective
22	Jing Pan	2024	1,685	530	100	1,055	Yes	Yes	No	1	China	Retrospective
23	Xiaocong Lin	2024	930	545	143	242	No	Yes	Yes	3	China	Retrospective
24	Sung Hye Kong	2024	1709	Unspecified	Unspecified	495	Yes	Yes	Yes	2	South Korea	Retrospective
25	Min Su Park	2024	1,122	831	291	NA	Yes	No	No	1	South Korea	Retrospective
26	Xiaoqing Yuan	2023	222	155	67	NA	Yes	No	No	1	China	Retrospective
27	Yu-Pin Chen	2024	1730	1,430	300	NA	No	No	No	1	Taiwan, China	Retrospective
28	Shigeng Wang	2024	2,274	1,660	267	347	No	No	No	1	China	Retrospective
29	Yu-Hsuan Lai	2024	412	Unspecified	Unspecified	NA	No	No	No	1	Taiwan, China	Retrospective
30	Shigeng Wang	2023	442	239	103	100	No	Yes	No	1	China	Retrospective
31	Jing Pan	2024	1,048	530	100	418	No	No	No	1	China	Retrospective
32	Tao Peng	2023	1,219	703	176	340	No	Yes	Yes	3	China	Retrospective
33	Yaling Pan	2023	1,175	195	698	282	No	Yes	Yes	Unknown	China	Retrospective
34	Keisuke Uemura	2023	857	315	542	NA	Yes	Yes	Yes	3	Japan	Retrospective
35	Xinyi Niu	2023	500	300	100	100	Yes	No	No	1	China	Retrospective
36	Amara Tariq	2023	4,406	3,525	881	NA	Yes	Yes	Yes	4	United States	Retrospective model development, prospective validation
37	Yung-Chieh Chen	2023	593	Unspecified, total 197		396	Yes	Yes	No	1	Taiwan, China	Retrospective
38	Jingnan Cui	2023	278	208	70	NA	Yes	Yes	No	1	China	Retrospective
39	Ronnie Sebro	2022	394	274	120	NA	Yes	No	No	1	United States	Retrospective
40	E Biamonte	2022	240	180	60	NA	Yes	No	No	1	Italy	Retrospective
41	Zhihao Xue	2022	133	Unspecified	Unspecified	NA	Yes	No	No	1	China	Retrospective
42	Ronnie Sebro	2022	196	96	100	NA	Yes	No	No	1	United States	Retrospective
43	Liyu Liu	2022	2,210	1768	442	NA	Yes	No	Yes	2	China	Retrospective
44	Miso Jang	2022	14,115	9,825	3,078	1,212	No	Yes	No	1	South Korea	Retrospective
45	Tomi Nissinen	2021	3,523	Unspecified	Unspecified	574	Yes	Yes	Yes	2	Finland	Retrospective model development, secondary analysis of prospective data for model validation
46	Yaling Pan	2020	574	160	40	374	No	No	No	1	China	Retrospective
47	Samir D Mehta	2019	307	246	61	NA	Yes	No	No	1	United States	Retrospective
48	A Valentinitsch	2019	154	0.8	0.2	NA	Yes	No	No	1	Germany	Retrospective
49	Chen-I Hsieh	2021	41,898	12,940	23,339	5,619	Yes	Yes	No	1	Taiwan, China	Retrospective
50	Qifei Dong	2022	4,416	3,413	379	669	No	No	Yes	6	United States	Retrospective
51	İlkay Yıldız Potter	2024	505	455	50	NA	No	Yes	No	1	United States	Retrospective
52	Jun Tang	2025	1880	1,184	296	400	Yes	Yes	Yes	2	China	Retrospective
53	Jaewon Kim	2025	14,502	10,152	1,450	2,900	No	No	No	1	South Korea	Retrospective
54	Amany M. Sarhan	2024	1947*	1558*	389*	NA	No	No	No	NA (public data)	NA (multi-region)	Retrospective
55	Xiaoling Zheng	2024	449	344	105	NA	No	No	No	1	China	Retrospective
56	Candan Güngör	2025	140	Unspecified	Unspecified	Unspecified	Yes	No	No	1	Turkey	Retrospective
57	Fatih Erdem	2024	140	90%	10%	NA	Yes	No	No	1	Turkey	Retrospective

*Represents image-level quantity; NA, not applicable.

In terms of study design, retrospective analyses were overwhelmingly predominant. Only three studies incorporated prospective elements or validation (42, 65, 84), indicating that current evidence remains largely built on retrospective cohort designs.

#### 3.2.2 Population characteristics

The included studies exhibited heterogeneity in population characteristics. Sample sizes varied widely, ranging from 109 to 75,524 participants, with a median of approximately 900. Notably, a study reported only the number of images rather than individual participants, reflecting inconsistencies in reporting practices (70). Most studies included both sexes, but females were consistently overrepresented, comprising approximately 60–65% of study populations on average. A small number of studies (*n* = 4) focused exclusively on women (31, 43, 58, 81), while one study included only male participants (79). This female predominance aligns with the higher prevalence of osteoporosis in women.

Age inclusion criteria also varied considerably, with lower thresholds ranging from ≥18 to ≥65 years, although most studies adopted thresholds of ≥40 or ≥50 years. This variability reflects a lack of standardization in target populations and may influence model performance. In particular, inclusion of younger, lower-risk individuals may dilute disease prevalence and affect model discrimination, whereas higher thresholds may improve screening efficiency but could miss early-stage disease.

Considerable variability was also observed in the clinical context of the study populations. Broadly, three categories could be identified: asymptomatic individuals undergoing routine health examinations, patients with specific underlying conditions (e.g., breast cancer (31), adrenal adenoma (34), degenerative spinal disorders (32, 49), or hip fractures (71)), and outpatient or emergency populations. This diversity reflects the opportunistic nature of screening, where imaging acquired for diverse clinical indications is repurposed for osteoporosis risk assessment. However, it also introduces important challenges, as differences in underlying disease status and clinical context may influence bone density distribution and imaging characteristics, potentially limiting model generalizability and necessitating domain adaptation across populations. Table 2 provides a detailed summary of these population characteristics.

**Table 2:** Summary of population characteristics.

Study ID	First author	Female	Male	Age range	Average age	Population	Gender	Inclusion age threshold
1	Wei L.	823	483	55–94	63.96 ± 7.92	Healthy individuals	Both sexes	≥55
2	Birtolo M. F.	109	0	27–85	61.1 ± 11.3	Breast cancer patients	Females only	>18
3	Hiyama A.	74	49	≥50	71.9 ± 8.3	Patients with degenerative spinal disorders	Both sexes	≥50
4	Ho U-C.	170	48	≥50	72.69 ± 9.96	Lumbar spine disease patients	Both sexes	≥50
5	Acosta-Batlle J.	1706	187	Not mentioned	72.3 ± 11.3	Healthy individuals	Both sexes	Unspecified (Average>72)
6	Jiyoung Song	192	191	≥40	59.8 ± 11.1	Healthy individuals	Both sexes	≥40
7	Li Y.	2,792	1,513	30–89	Not mentioned	Healthy individuals	Both sexes	>30
8	Cheng Jia	67	94	36–60	Median age 53	Patients with adrenal adenoma	Both sexes	Unspecified (observed range 36–60)
9	Li Y.	605	562	Not mentioned	Not mentioned	Patients undergoing LDCT for lung cancer screening	Both sexes	Unspecified
10	Park J.	38,767	36,757	40–100	Not mentioned	Health check population and clinical patients	Both sexes	≥40
11	Tang J.	1,046	734	50–99	69.46 ± 10.33	Patients undergoing chest X-ray	Both sexes	≥50
12	Ren Q.	237	272	30–80	Not mentioned	Patients undergoing abdominal CT	Both sexes	≥30
13	Li Y.	591	396	30–89	Not mentioned	Patients undergoing LDCT for lung cancer screening	Both sexes	≥30
14	Fanelli F.	301	0	≥50	Not mentioned	Postmenopausal women	Females only	≥50
15	Du C.	239	217	50–91	67.80 ± 8.18	Patients undergoing low-dose abdominal CT	Both sexes	≥50
16	Wu Y.	1,540	1772	Not mentioned	Not mentioned	Healthy individuals	Both sexes	Unspecified
17	Farid Gharehmohammadi	767	140	≥50	66.9 ± 9.0	Healthy individuals	Both sexes	≥50
18	Chengbin Huang	Unspecified	Unspecified	≥50	Not mentioned	Patients undergoing chest CT	Both sexes	≥50
19	Qiye Cheng	86	128	Not mentioned	Not mentioned	Patients undergoing abdominal DEsCT	Both sexes	Unspecified
20	Fabio Galbusera	313	116	33–92	Median age 72	Patients with degenerative spinal disorders	Both sexes	Unspecified (observed range 33–92)
21	Heejun Park	260	160	Not mentioned	60.3 ± 15.4	Patients undergoing chest/abdominal/lumbar spine CT	Both sexes	Unspecified
22	Jing Pan	810	875	20–92	Not mentioned	Healthy individuals	Both sexes	≥18
23	Xiaocong Lin	647	283	≥18	Not mentioned	Patients undergoing chest CT	Both sexes	≥18
24	Sung Hye Kong	1,229	480	50–80	Not mentioned	Patients undergoing abdominal CT	Both sexes	50–80
25	Min Su Park	989	133	≥50	Not mentioned	Patients undergoing abdominopelvic CT	Both sexes	≥50
26	Xiaoqing Yuan	148	74	50–92	66.4 ± 9.4	Patients undergoing abdominal CT	Both sexes	≥50
27	Yu-Pin Chen	1,332	398	≥50	72.4 ± 11.1	Patients undergoing hip radiography	Both sexes	≥50
28	Shigeng Wang	1,009	1,265	Not mentioned	Not mentioned	Patients undergoing chest CT	Both sexes	Unspecified
29	Yu-Hsuan Lai	412	0	Not mentioned	61.5 ± 10.1	Female breast cancer patients	Females only	Unspecified
30	Shigeng Wang	178	264	Not mentioned	61.86 ± 11.38	Patients undergoing low-dose chest CT (LDCT)	Both sexes	Unspecified
31	Jing Pan	443	605	20–92	51.19 ± 14.35	Healthy individuals	Both sexes	≥18
32	Tao Peng	Unspecified	Unspecified	Not mentioned	Not mentioned	Patients undergoing opportunistic CT (chest, abdomen, lumbar spine) at multiple centers	Both sexes	Unspecified
33	Yaling Pan	402	773	32–88	Not mentioned	Patients undergoing chest LDCT	Both sexes	Unspecified (observed range 32–88)
34	Keisuke Uemura	668	189	Not mentioned	70.0 ± 16.8	Patients undergoing hip surgery	Both sexes	Unspecified
35	Xinyi Niu	Unspecified	Unspecified	39–90	66.03 ± 9.71	General population	Both sexes	Unspecified (observed range 39–90)
36	Amara Tariq	2,878	1,528	Not mentioned	66.9 ± 9.2	Patients undergoing abdominopelvic CT	Both sexes	Unspecified
37	Yung-Chieh Chen	Unspecified	Unspecified	Not mentioned	Not mentioned	Healthy individuals	Both sexes	Unspecified
38	Jingnan Cui	Unspecified	Unspecified	Not mentioned	Not mentioned	Postmenopausal women (spine); healthy and osteoporotic fracture patients (calcaneus)	Spine: females only; calcaneus: unspecified	≥50 (Spine)
39	Ronnie Sebro	286	108	50–87	62.1 ± 7.4	Patients undergoing abdominopelvic CT	Both sexes	≥50
40	E Biamonte	110	130	≥18	60.4 ± 15.4	Patients undergoing lumbar spine CT	Both sexes	≥18
41	Zhihao Xue	92	41	31–94	65.45 ± 9.82	Patients with low back pain undergoing lumbar spine CT	Both sexes	Unspecified (observed range 31–94)
42	Ronnie Sebro	173	23	50–88	64.9 ± 8.7	Patients undergoing wrist CT	Both sexes	≥50
43	Liyu Liu	Unspecified	Unspecified	>40	Not mentioned	Health check population and clinical patients	Both sexes	>40
44	Miso Jang	11,811	2,304	40–90	Not mentioned	Health check population (training/internal test) and clinical patients (external test)	Both sexes	>40
45	Tomi Nissinen	3,408	115	20–96	Not mentioned	Postmenopausal women and clinical patients	Both sexes	Unspecified
46	Yaling Pan	Unspecified	Unspecified	50–88	62.6 ± 7.6	Patients undergoing LDCT	Both sexes	≥50
47	Samir D Mehta	202	105	Not mentioned	Not mentioned	Patients undergoing DXA	Both sexes	Unspecified (observed range 67–71)
48	A Valentinitsch	51	103	39–88	64.6 ± 8.5	Cancer patients	Both sexes	>38
49	Chen-I Hsieh	18,466	4,873	40–90	Not mentioned	Patients undergoing pelvic/spine X-ray	Both sexes	40–90
50	Qifei Dong	0	4,416	≥65	73.7	Community-dwelling older men	Males only	≥65
51	İlkay Yıldız Potter	186	130	18–100	Not mentioned	Emergency or outpatient patients	Both sexes	Unspecified (observed range 18–100)
52	Jun Tang	1,104	776	≥50	Not mentioned	Clinical patients	Both sexes	≥50
53	Jaewon Kim	14,502	0	20–88	55.97 ± 9.69	Female health check population	Females only	Unspecified (observed range 20–88)
54	Amany M. Sarhan	Unspecified	Unspecified	Not mentioned	Not mentioned	Patients undergoing knee radiography	Unspecified	Unspecified
55	Xiaoling Zheng	300	149	Not mentioned	Not mentioned	Hip fracture patients	Both sexes	Unspecified
56	Candan Güngör	124	16	33–91	Not mentioned	Clinical patients	Both sexes	Unspecified (observed range 33–91)
57	Fatih Erdem	132	8	Not mentioned	65.32 ± 8.50	Clinical patients	Both sexes	Unspecified

DXA, dual-energy X-ray absorptiometry.

Overall, the marked heterogeneity in study design, sample size, demographic composition, and clinical context should be carefully considered when interpreting subsequent findings on imaging modalities and AI model performance.

### 3.3 Modeling and imaging characteristics: predominance of automated deep learning

#### 3.3.1 Adoption of automated imaging ROI

Table 3 summarizes the key ROI characteristics of the included studies. CT served as the primary data source for AI-driven opportunistic osteoporosis screening, being employed in 38 studies and encompassing a range of acquisition protocols, including conventional, low-dose, non-contrast, and contrast-enhanced multi-detector CT. X-ray-based approaches were also frequently utilized (*n* = 15). In contrast, MRI was only rarely applied (*n* = 3) (33, 49, 86), consistent with its limited role in the quantitative assessment of bone mineral density. Despite its status as the clinical reference standard, DXA was incorporated into opportunistic screening workflows in 2 studies (78, 84). In these cases, DXA data were analyzed in an automated manner without requiring additional imaging, reflecting efforts to extend its utility beyond traditional diagnostic settings.

**Table 3:** Summary of region of interest characteristics.

Study ID	First author	Modality	ROI	ROI category	ROI segmentation
1	Wei L.	CT	T10, T11, T12, and L1 vertebrae	Spinal vertebrae	AI-based automatic segmentation
2	Birtolo M. F.	CT	L1–L5 vertebrae	Spinal vertebrae	AI-based automatic segmentation
3	Hiyama A.	CT	L1–L4 vertebrae	Spinal vertebrae	Manual delineation
4	Ho U-C.	MRI	L1–L4 vertebrae	Spinal vertebrae	Manual delineation
5	Acosta-Batlle J.	X-ray	Proximal femur	Hip/proximal femur	Automatic segmentation and manual delineation
6	Jiyoung Song	CT	Thoracolumbar vertebrae	Spinal vertebrae	AI-based automatic segmentation
7	Li Y.	LDCT	T12–L2 vertebrae	Spinal vertebrae	AI-based automatic segmentation
8	Cheng Jia	CT	Thoracolumbar vertebrae	Spinal vertebrae	Manual delineation
9	Li Y.	LDCT	T12–L2 vertebrae	Spinal vertebrae	AI-based automatic segmentation
10	Park J.	X-ray	Chest	Whole image/non-precise segmentation	AI-based automatic segmentation
11	Tang J.	X-ray	Chest	Whole image/non-precise segmentation	AI-based automatic segmentation
12	Ren Q.	CT	L1–L3 vertebrae	Spinal vertebrae	Manual delineation
13	Li Y.	LDCT	T12–L2 vertebrae	Spinal vertebrae	AI-based automatic segmentation
14	Fanelli F.	X-ray	Posterior mandible region	Whole image/non-precise segmentation	Manual delineation
15	Du C.	LDCT	Proximal femur	Hip/proximal femur	AI-based automatic segmentation
16	Wu Y.	LDCT	T12–L2 vertebrae	Spinal vertebrae	AI-based automatic segmentation
17	Farid Gharehmohammadi	X-ray	Feet or ankle	Whole image/non-precise segmentation	AI-based automatic segmentation
18	Chengbin Huang	CT	T12 vertebra and surrounding skeletal muscles	Spinal vertebrae	Manual delineation
19	Qiye Cheng	CT	L1–L3 vertebrae	Spinal vertebrae	Manual delineation
20	Fabio Galbusera	MRI, X-ray	L1–L4 vertebrae	Spinal vertebrae	AI-based automatic segmentation
21	Heejun Park	CT	L1 and L2 vertebrae	Spinal vertebrae	AI-based automatic segmentation
22	Jing Pan	CT	L1 and L2 vertebrae	Spinal vertebrae	AI-based automatic segmentation
23	Xiaocong Lin	CT	T11 or T12 vertebra	Spinal vertebrae	Semi-Automatic segmentation
24	Sung Hye Kong	CT	T12–L4 vertebrae	Spinal vertebrae	AI-based automatic segmentation
25	Min Su Park	CT	Proximal femur	Hip/proximal femur	AI-based automatic segmentation
26	Xiaoqing Yuan	CT	Left proximal femur	Hip/proximal femur	AI-based automatic segmentation
27	Yu-Pin Chen	X-ray	Proximal femur	Hip/proximal femur	AI-based automatic segmentation
28	Shigeng Wang	CT	T10–T12 vertebrae	Spinal vertebrae	AI-based automatic segmentation
29	Yu-Hsuan Lai	CT	L1 vertebra	Spinal vertebrae	Manual delineation
30	Shigeng Wang	LDCT	T10–T12 vertebrae	Spinal vertebrae	AI-based automatic segmentation
31	Jing Pan	CT	L1 and L2 vertebrae	Spinal vertebrae	AI-based automatic segmentation
32	Tao Peng	CT	T12–L2 or L1–L3 vertebrae	Spinal vertebrae	AI-based automatic segmentation
33	Yaling Pan	LDCT	T11–L2 vertebrae	Spinal vertebrae	AI-based automatic segmentation
34	Keisuke Uemura	CT	Proximal femur	Hip/proximal femur	AI-based automatic segmentation
35	Xinyi Niu	LDCT	T12–L2 vertebrae	Spinal vertebrae	AI-based automatic segmentation
36	Amara Tariq	CT	L3 vertebra, hip, and lumbar region	Multi-site/combination	AI-based automatic segmentation
37	Yung-Chieh Chen	LDCT	L1–L4 vertebrae	Spinal vertebrae	AI-based automatic segmentation
38	Jingnan Cui	X-ray	L4 vertebra and calcaneus	Multi-site/combination	Semi-Automatic segmentation
39	Ronnie Sebro	CT	L1–L4 vertebrae, pelvis and proximal femur	Multi-site/combination	Semi-Automatic segmentation
40	E Biamonte	CT	L1–L5 vertebrae	Spinal vertebrae	Manual delineation
41	Zhihao Xue	CT	L1–L4 vertebrae	Spinal vertebrae	AI-based automatic segmentation
42	Ronnie Sebro	CT	Radius, ulna, carpals, metacarpals	Whole image/non-precise segmentation	Manual delineation
43	Liyu Liu	CT	L1–L4 vertebrae	Spinal vertebrae	Manual delineation
44	Miso Jang	X-ray	Chest	Whole image/non-precise segmentation	AI-based automatic segmentation
45	Tomi Nissinen	DXA	L1–L4 vertebrae	Spinal vertebrae	AI-based automatic segmentation
46	Yaling Pan	LDCT	T12–L2 vertebrae	Spinal vertebrae	AI-based automatic segmentation
47	Samir D Mehta	DXA	L1–L4 vertebrae	Spinal vertebrae	No segmentation (using DEXA report data)
48	A Valentinitsch	MDCT	T12–L4 vertebrae	Spinal vertebrae	AI-based automatic segmentation
49	Chen-I Hsieh	X-ray	L1–L4 vertebrae and proximal femur	Multi-site/combination	AI-based automatic segmentation
50	Qifei Dong	X-ray	Single spine vertebra	Spinal vertebrae	Manual delineation
51	İlkay Yıldız Potter	X-ray	hip	Whole image/non-precise segmentation	No segmentation, whole image input
52	Jun Tang	X-ray	Chest	Whole image/non-precise segmentation	No segmentation, whole image input
53	Jaewon Kim	X-ray	Chest	Whole image/non-precise segmentation	No segmentation, whole image input
54	Amany M. Sarhan	X-ray	knee	Whole image/non-precise segmentation	No segmentation, whole image input
55	Xiaoling Zheng	CT	T11 or T12 vertebra	Spinal vertebrae	Semi-Automatic segmentation
56	Candan Güngör	CT	Clivus	Clivus	Semi-Automatic segmentation
57	Fatih Erdem	MRI	lumbar	Spinal vertebrae	Manual delineation

CT, Computed Tomography; LDCT, Low-Dose Computed Tomography; MRI, Magnetic Resonance Imaging; ROI, Region of Interest; AI, Artificial intelligence; DXA, dual-energy X-ray absorptiometry.

From an anatomical perspective, the spine emerged as the predominant ROI, featuring in 35 studies. Analyses typically focused on vertebral levels such as L1-L4 or T12-L2, given their strong correlation with bone mineral density and their suitability for standardized assessment. Other studies targeted the hip or proximal femur (*n* = 6) (30, 44, 54–56, 63) or adopted multisite strategies integrating multiple skeletal regions (*n* = 4).

More recently, opportunistic screening has been extended to the craniofacial region, with two included studies leveraging clivus-based CT radiomic features (72) and mandibular radiographs (43), respectively. These emerging anatomical targets carry considerable clinical relevance, as head and maxillofacial imaging is routinely performed in emergency, dental, and geriatric care, generating a vast, currently underutilized resource for bone health assessment. While the clivus approach exploits feature already present in standard head CTs, mandibular radiographs offer a complementary, low-radiation modality that could further broaden population-level screening reach. Notably, ten studies dispensed with predefined ROIs altogether, instead leveraging whole-image inputs.

Regarding ROI extraction, deep learning-based automatic segmentation was the most common approach (*n* = 34), enabling scalable and fully automated pipelines. Manual (*n* = 12) (32–34, 41, 43, 47, 48, 58, 74, 77, 79, 82) and semi-automated (*n* = 4) (52, 67, 71, 76) methods were less frequent. Studies that used whole image inputs did not require explicit ROI segmentation, further simplifying preprocessing workflows. Recent investigations have increasingly emphasized fully automated segmentation strategies, transitioning from manual delineation to deep learning-based methods (e.g., nnU-Net, DeepLab, DeepCatch), thereby substantially reducing labor demands. While such approaches may better align with real-world clinical deployment, they may also diminish anatomical interpretability.

Together, these patterns underscore a broader shift toward automation and streamlined model inputs, a trajectory that extends to subsequent stages of feature extraction and model development.

#### 3.3.2 Deep learning as the dominant modeling framework

Table 4 summarizes the feature extraction methods and prediction algorithms. Feature extraction strategies could be broadly categorized into three groups. Deep learning-based approaches were the most prevalent (*n* = 29), enabling end-to-end representation learning directly from imaging data. Handcrafted radiomics features were employed in 18 studies, typically involving the extraction of texture and shape descriptors followed by feature selection procedures. A smaller subset of studies relied on conventional CT attenuation metrics, such as Hounsfield units (HU) (*n* = 5). In addition, two studies combined deep learning-derived features with radiomics, while one study utilized quantitative outputs from DXA as numerical inputs.

**Table 4:** Characteristics of prediction models.

Study ID	First author	Feature extraction	Prediction model algorithm	Model type (Combined imaging and clinical vs. imaging-only)	Model comparison performed
1	Wei L.	Deep Learning Features	Conventional statistical model	Imaging-only	No
2	Birtolo M. F.	Handcrafted Radiomics	Conventional statistical model and machine learning	Combined model	Yes
3	Hiyama A.	HU Measurements	Conventional statistical model and machine learning	Combined model	No
4	Ho U-C.	Deep Learning Features	Deep learning model	Imaging-only	No
5	Acosta-Batlle J.	Deep Learning Features	Deep learning model	Imaging-only	No
6	Jiyoung Song	Deep Learning Features	Traditional machine learning	Combined model	Yes
7	Li Y.	Deep Learning Features	Deep learning model	Imaging-only	No
8	Cheng Jia	Handcrafted Radiomics	Conventional statistical model	Combined model	Yes
9	Li Y.	Deep Learning Features	Deep learning model	Imaging-only	No
10	Park J.	Deep Learning Features	Deep learning model	Imaging-only	No
11	Tang J.	Deep Learning Features	Deep learning model	Combined model	Yes
12	Ren Q.	Handcrafted Radiomics	Traditional machine learning	Imaging-only	No
13	Li Y.	Deep Learning Features	Deep learning model	Imaging-only	No
14	Fanelli F.	Deep Learning Features and Handcrafted Radiomics	Deep learning model	Imaging-only	No
15	Du C.	Handcrafted Radiomics	Traditional machine learning	Imaging-only	No
16	Wu Y.	Deep Learning Features	Deep learning model	Imaging-only	No
17	Farid Gharehmohammadi	Deep Learning Features	Deep learning model	Imaging-only	No
18	Chengbin Huang	Deep Learning Features	Deep learning model	Imaging-only	Yes
19	Qiye Cheng	Handcrafted Radiomics	Traditional machine learning	Imaging-only	No
20	Fabio Galbusera	Handcrafted Radiomics	Deep learning and traditional machine learning	Combined model	Yes
21	Heejun Park	Deep Learning Features	Deep learning model	Imaging-only	No
22	Jing Pan	Deep Learning Features and Handcrafted Radiomics	Deep learning model	Combined model	Yes
23	Xiaocong Lin	Handcrafted Radiomics	Traditional machine learning	Combined model	Yes
24	Sung Hye Kong	Deep Learning Features	Deep learning model	Combined model	Yes
25	Min Su Park	Handcrafted Radiomics	Traditional machine learning	Combined model	No
26	Xiaoqing Yuan	Handcrafted Radiomics	Conventional statistical model	Imaging-only	No
27	Yu-Pin Chen	Deep Learning Features	Deep learning model	Combined model	No
28	Shigeng Wang	Deep Learning Features	Deep learning model	Imaging-only	No
29	Yu-Hsuan Lai	Handcrafted Radiomics	Conventional statistical model	Combined model	Yes
30	Shigeng Wang	Handcrafted Radiomics	Traditional machine learning	Imaging-only	No
31	Jing Pan	Deep Learning Features	Deep learning model	Imaging-only	No
32	Tao Peng	Deep Learning Features	Deep learning model	Imaging-only	No
33	Yaling Pan	HU Measurements	Conventional statistical model	Imaging-only	No
34	Keisuke Uemura	HU Measurements	Deep learning model	Imaging-only	No
35	Xinyi Niu	Deep Learning Features	Deep learning model	Imaging-only	No
36	Amara Tariq	Deep Learning Features	Deep learning, traditional machine learning and conventional statistical model	Combined model	Yes
37	Yung-Chieh Chen	Handcrafted Radiomics	Traditional machine learning	Imaging-only	No
38	Jingnan Cui	Handcrafted Radiomics	Traditional machine learning	Imaging-only	No
39	Ronnie Sebro	HU Measurements	Conventional statistical model and machine learning	Combined model	No
40	E Biamonte	Handcrafted Radiomics	Traditional machine learning	Imaging-only	No
41	Zhihao Xue	Handcrafted Radiomics	Traditional machine learning	Imaging-only	No
42	Ronnie Sebro	HU Measurements	Traditional machine learning	Imaging-only	Yes
43	Liyu Liu	Handcrafted Radiomics	Conventional statistical model and machine learning	Combined model	Yes
44	Miso Jang	Deep Learning Features	Deep learning model	Imaging-only	No
45	Tomi Nissinen	Deep Learning Features	Deep learning model	Imaging-only	No
46	Yaling Pan	Deep Learning Features	Deep learning model	Imaging-only	No
47	Samir D Mehta	DXA-derived Numerical Features	Traditional machine learning	Imaging-only	No
48	A Valentinitsch	Handcrafted Radiomics	Traditional machine learning	Imaging-only	No
49	Chen-I Hsieh	Deep Learning Features	Deep learning model	Imaging-only	No
50	Qifei Dong	Deep Learning Features	Deep learning model	Imaging-only	No
51	İlkay Yıldız Potter	Deep Learning Features	Deep learning model	Imaging-only	No
52	Jun Tang	Deep Learning Features	Deep learning model	Imaging-only	No
53	Jaewon Kim	Deep Learning Features	Deep learning model	Imaging-only	No
54	Amany M. Sarhan	Deep Learning Features	Deep learning model	Imaging-only	No
55	Xiaoling Zheng	Handcrafted Radiomics	Traditional machine learning	Combined model	Yes
56	Candan Güngör	Handcrafted Radiomics	Traditional machine learning	Imaging-only	No
57	Fatih Erdem	Handcrafted Radiomics	Traditional machine learning	Imaging-only	No

Model type: imaging-only, model built with imaging features alone; combined, model built with imaging plus clinical features. Model comparison performed: yes/no indicates whether the study directly compared the predictive performance of the imaging-only and combined models. DXA, dual-energy X-ray absorptiometry.

In terms of predictive frameworks, deep learning models were most frequently adopted (*n* = 29), followed by traditional machine learning approaches (*n* = 15), including support vector machines (SVM), random forests (RF), and logistic regression (LR). The remaining studies employed conventional statistical models or hybrid methodologies. A clear temporal trend was observed: earlier studies (2019–2022) more commonly relied on HU-based features or radiomics coupled with machine learning, whereas studies published from 2023 onward predominantly adopted deep learning architectures.

Widely used deep learning architectures included convolutional neural networks (e.g., ResNet, DenseNet, EfficientNet, Inception, and VGG) and segmentation frameworks such as U-Net-based models. These architectures often supported end-to-end pipelines, spanning from ROI segmentation to downstream tasks such as bone mineral density estimation, osteoporosis classification, and fracture risk prediction.

Notably, several studies published in 2025 reported fine-tuning of large pretrained models, including OpenCLIP, DINOv2, and CheXagent, highlighting an emerging shift toward foundation model-based paradigms (69, 70, 81).

Compared with radiomics-based approaches, deep learning models generally offer improved reproducibility and reduced reliance on manual intervention. Nonetheless, radiomics retains value in terms of interpretability, as extracted features can be directly mapped to specific image characteristics. Hybrid strategies, such as combining deep learning-based segmentation with radiomics-driven classification, were also observed, aiming to balance automation with interpretability.

Overall, the field is transitioning toward increasingly automated modeling frameworks, with deep learning assuming a central role in both feature extraction and predictive modeling.

#### 3.3.3 The underestimated potential of clinical features in osteoporosis screening

The majority of included studies developed imaging-only models (*n* = 39), whereas a smaller subset (*n* = 16) incorporated both imaging and clinical variables. Notably, no study relied exclusively on clinical features. Among studies adopting a multimodal approach, commonly included clinical variables comprised age, sex, body mass index, and laboratory parameters. Of these, 13 studies explicitly compared the performance of imaging-only models with that of combined models, yielding largely consistent findings (Table 4).

Overall, the integration of clinical data conferred modest yet measurable improvements in predictive performance, as reflected by increases in metrics such as the area under the curve (AUC) and overall accuracy. However, several studies reported negligible or no incremental benefit from including clinical variables (38, 52, 71), particularly in scenarios where imaging models had already captured rich, high-dimensional representations of the underlying pathology. For example, models leveraging vertebral imaging and body composition features demonstrated strong predictive performance without appreciable gains from additional clinical inputs (38). The model comparison information is reported in Table 4.

These findings should not be taken to imply that clinical features lack predictive value. A very recent study demonstrated that a model relying solely on 35 routine clinical variables achieved an accuracy of 83.94% for opportunistic osteoporosis screening across three hospitals (87). The model combined Transformer, Mamba, and a FeatureBake Block with a CACM module, and SHAP analyses identified hemoglobin, age, and weight as the most influential predictors, linking model decisions to clear physiological rationales. This evidence highlights that, even without imaging, clinical variables can carry substantial discriminative power. The limited incremental gains observed in earlier imaging-dominant settings may therefore reflect ceiling effects rather than an intrinsic weakness of clinical data.

Collectively, these findings suggest that the modest gains from adding clinical variables in prior imaging-based studies likely reflect ceiling effects of high-performance imaging models, rather than an inherent lack of predictive value in clinical data.

### 3.4 Quality assessment: deficits in external validation as a primary source of bias

To systematically evaluate the quality of the included literature, we employed two frameworks: the phase classification criteria and the Prediction Model Risk of Bias Assessment Tool (PROBAST). The phase classification criteria stratify studies according to sample size, study design (retrospective vs. prospective), and validation status (none, internal, or external). Given that all studies enrolled more than 100 participants and were predominantly retrospective in design, 55 were classified as Phase II, whereas the remaining two studies involving prospective data collection or validation were accordingly designated Phase III (Table 5). Inter-reviewer agreement was 98.25% (56/57), with a Kappa of 0.66. The modest Kappa value despite near-perfect observed agreement is attributable to the severe class imbalance in the data, which inflates the expected agreement and exerts a strong downward correction on Kappa. The wide confidence interval further reflects the small number of studies and the extreme prevalence of Phase II ratings. Nevertheless, the highly observed agreement indicates that inter-reviewer reliability is practically acceptable.

**Table 5:** Summary of the quality of included studies.

Study ID	First author	Phase	ROB
1	Wei L.	II	High
2	Birtolo M. F.	II	High
3	Hiyama A.	II	High
4	Ho U-C.	II	High
5	Acosta-Batlle J.	II	High
6	Jiyoung Song	II	High
7	Li Y.	II	Low
8	Cheng Jia	II	High
9	Li Y.	II	Low
10	Park J.	II	Low
11	Tang J.	II	High
12	Ren Q.	II	Low
13	Li Y.	II	Low
14	Fanelli F.	II	Low
15	Du C.	II	High
16	Wu Y.	II	Low
17	Farid Gharehmohammadi	II	High
18	Chengbin Huang	II	Low
19	Qiye Cheng	II	High
20	Fabio Galbusera	II	High
21	Heejun Park	II	Low
22	Jing Pan	II	Low
23	Xiaocong Lin	II	Low
24	Sung Hye Kong	II	Low
25	Min Su Park	II	High
26	Xiaoqing Yuan	II	High
27	Yu-Pin Chen	II	High
28	Shigeng Wang	II	High
29	Yu-Hsuan Lai	II	High
30	Shigeng Wang	II	Low
31	Jing Pan	II	High
32	Tao Peng	II	Low
33	Yaling Pan	II	Low
34	Keisuke Uemura	II	Low
35	Xinyi Niu	II	High
36	Amara Tariq	III	Low
37	Yung-Chieh Chen	II	Low
38	Jingnan Cui	II	Low
39	Ronnie Sebro	II	High
40	E Biamonte	II	High
41	Zhihao Xue	II	High
42	Ronnie Sebro	II	High
43	Liyu Liu	II	High
44	Miso Jang	II	Low
45	Tomi Nissinen	III	Low
46	Yaling Pan	II	High
47	Samir D Mehta	II	High
48	A Valentinitsch	II	High
49	Chen-I Hsieh	II	Low
50	Qifei Dong	II	High
51	İlkay Yıldız Potter	II	Low
52	Jun Tang	II	Low
53	Jaewon Kim	II	High
54	Amany M. Sarhan	II	High
55	Xiaoling Zheng	II	High
56	Candan Güngör	II	High
57	Fatih Erdem	II	High

Phase, phase classification criteria; ROB, risk of bias.

**Figure 2:**
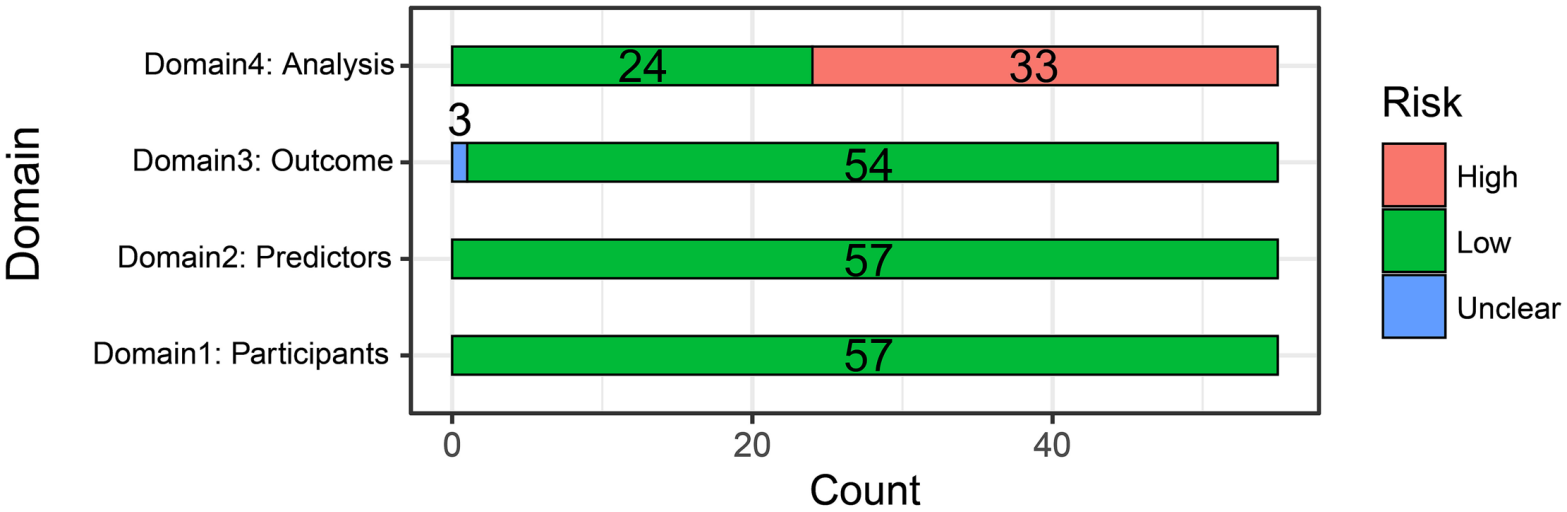
Evaluation of risk of bias of included studies.

Risk of bias was further assessed using PROBAST across four domains: Participants, Predictors, Outcome, and Analysis. As this review focuses on model development studies, the absence of external validation is, by PROBAST standards, indicative of high risk of bias. Accordingly, 25 studies were judged to be at high risk. Domain-specific assessments are summarized in Table 5 and Figure 2.

Validation designs varied substantially across studies, particularly in data partitioning, use of resampling methods, and implementation of external validation. Most studies adopted simple train-test splits, typically using ratios such as 70/30 or 80/20. In several cases, validation and testing were not clearly distinguished, and some lacked an independent test set, relying instead on internal validation or cross-validation. Approximately half of the studies employed cross-validation, primarily in the context of limited sample sizes, as an attempt to enhance robustness through resampling. While this approach can reduce overfitting, it does not replace the need for independent external validation.

Only 20 studies performed external validation. Among these, a small proportion used truly independent datasets from different institutions or populations, including a limited number of multicenter studies. In many cases, external validation was conducted within similar geographic or institutional settings, which may limit the assessment of model performance across diverse populations and imaging conditions. Truly international and multi-ethnic validation was rare, with only isolated examples (37).

Sample size was not consistently associated with a stronger study design. Although some large studies (>10,000 participants), such as Park (2025) (37) and Jang (2022) (80), included external validation, most studies with moderate sample sizes (1,000-5,000) did not, and smaller studies (<500) generally relied on internal validation alone. It is also important to note that large retrospective, single-center studies may still be affected by selection bias and site-specific imaging characteristics.

Overall, current studies show increasing but still limited use of external and multicenter validation. Notably, this review primarily focuses on model development studies. Compared with model validation studies, model development studies are inherently more difficult to validate across multiple centers due to external resource constraints. This is an intrinsic limitation of this research type. These factors should be considered when interpreting reported model performance, which is examined in the following section.

### 3.5 Model performance: variability across tasks and clinical endpoints

To enhance transparency and clinical applicability, we performed descriptive stratification of the 57 included studies by predicted outcome (osteoporosis diagnosis, osteopenia, and fracture risk), imaging modality, and model type (Table 6). Overall, CT was the dominant imaging modality (particularly for osteoporosis and osteopenia prediction), while deep learning approaches predominated across all outcome categories. Fracture risk prediction had the fewest studies and generally lower performance. Detailed stratification is presented in Table 6.

**Table 6:** Descriptive stratification of included studies and model performance by imaging modality and model type.

Predicted outcome	Imaging modality	Model type	No. of studies	No. with external validation (%)	Median AUC (Range)
Osteoporosis	CT	Deep Learning	13	7 (54%)	0.94 (0.82–1.0)
		Traditional ML/Hybrid	15	6 (40%)	0.91 (0.79–0.99)
	MRI	Deep Learning	1	0 (0%)	0.83
		Traditional ML/Hybrid	2	0 (0%)	0.86 (0.8–0.91)
	X-ray	Deep Learning	9	4 (44%)	0.93 (0.81–0.99)
		Traditional ML/Hybrid	2	2 (100%)	0.78 (0.77–0.79)
Osteopenia	CT	Deep Learning	13	8 (62%)	0.94 (0.69–1)
		Traditional ML/Hybrid	11	4 (36%)	0.94 (0.81–0.98)
	MRI	Traditional ML/Hybrid	1	0 (0%)	0.82
	X-ray	Deep Learning	2	1 (50%)	0.91 (0.87–0.95)
		Traditional ML/Hybrid	1	1 (100%)	0.72
Fracture Risk	CT	Deep Learning	1	1 (100%)	0.83
		Traditional ML/Hybrid	3	0 (0%)	0.83 (0.79–0.88)
	DXA	DXA-derived Numerical Features	2	1 (50%)	0.77 (0.63–0.91)
	X-ray	Deep Learning	3	2 (67%)	0.94 (0.82–0.99)

AUC, area under the curve; CT, computed tomography; MRI, magnetic resonance imaging; DXA, dual-energy X-ray absorptiometry; ML, machine learning.

#### 3.5.1 Performance variability in osteoporosis prediction

A total of 42 studies reported performance for osteoporosis prediction, comprising 45 models, as one study constructed separate models based on the T10, T11, T12, and L1 vertebrae (39). To compare the performance of these models on the same scale, we extracted the AUC values. Overall, model discrimination varied substantially, with AUC values ranging from 0.767 to 1.000. Notably, eight models achieved an AUC ≥ 0.98, including three reporting perfect discrimination (AUC = 1.000) (35, 36, 42). While these three studies employed patient-level splitting with external validation sets drawn from independent centers and scanners, perfect AUC values on external data remain highly implausible and should not be attributed merely to cohort size. A more likely explanation is some form of data leakage that cannot be fully discerned from the published methods. This concern is reinforced by the observation in Ref (33) that AUC values of 1.000 were achieved across all test sets, both internal and external, a pattern difficult to reconcile with genuine generalization. Accordingly, these perfect scores warrant extreme caution in interpretation.

Performance varied according to imaging modality and modeling strategy. As shown in Table 6, CT-based models constituted the majority of osteoporosis prediction studies, with deep learning models achieving a median AUC of 0.94 (range 0.82–1.00) and traditional machine learning (ML)/hybrid models a median of 0.91 (0.79–0.99). MRI-based models remained limited (*n* = 2), yielding more modest AUCs (0.80–0.86). Radiography (X-ray) studies showed strong performance in deep learning models (median AUC 0.93), though with fewer studies overall.

The two MRI-based models yielded relatively modest AUCs (0.80 and 0.83) (33, 49). However, recent work suggests that functional MRI sequences may offer a more promising direction. Erdem et al. (2024) applied radiomic feature extraction to ADC maps of lumbar vertebrae and combined the selected features with machine learning classifiers (86). A Naive Bayes model trained on five wavelet- and Laplacian-of-Gaussian-derived features achieved an AUC of 0.913, with 90.7% accuracy and 95% specificity. Because ADC values reflect microarchitectural changes in bone marrow, such as shifts in fat and water content, functional sequences like DWI and ADC mapping may capture information that conventional T1- and T2-weighted images cannot. Although this evidence comes from a single study with a modest sample size and requires external validation, it indicates that advanced MRI sequences represent a viable pathway for AI-driven opportunistic osteoporosis screening and merit further investigation (88).

Models using whole-image inputs without predefined regions of interest achieved strong performance (AUC: 0.82–0.99), albeit with reduced interpretability. In terms of feature extraction approaches, the deep learning-based approach (*n* = 27) demonstrated better performance (median AUC = 0.93) compared with handcrafted radiomics features (*n* = 12, median AUC = 0.90) and models based on Hounsfield unit measurements (*n* = 4, median AUC = 0.89).

#### 3.5.2 Task-dependent performance in osteopenia prediction

A total of 28 studies developed models for osteopenia prediction, comprising 31 models, including four vertebra-specific models (T10, T11, T12, and L1) from a single study (39). Overall discrimination was highly variable (AUC: 0.69–1.000), with a lower performance bound than that observed for osteoporosis prediction (0.69 vs. 0.767), indicating reduced separability for this intermediate phenotype. Despite this variability, a subset of models demonstrated excellent performance, with seven achieving AUC ≥ 0.98, including four with perfect discrimination (AUC = 1.000) (35, 36, 42).

Stratification by imaging modality (Table 6) revealed that CT remained the most common modality for osteopenia prediction as well. Deep learning models on CT achieved a median AUC of 0.94 (0.69–1.00), while traditional ML/hybrid models showed comparable performance (median 0.94). Radiography-based deep learning models also performed well (AUC 0.91), although data for MRI and other modalities were sparse.

Heterogeneity in task design may partly explain this variability. Some studies adopted binary classification (normal vs. osteopenia), whereas others implemented multiclass classification (normal/osteopenia/osteoporosis), the latter representing a more clinically relevant yet inherently more challenging task. Notably, the lowest AUC (0.69) was reported in a multiclass model based on the L1 vertebra, using DXA-defined thresholds (−2.5 < T-score <= − 1.0), underscoring the difficulty of discriminating osteopenia from adjacent categories.

Collectively, these findings suggest that osteopenia prediction is sensitive to task design and remains inherently more challenging despite the presence of high-performing models.

#### 3.5.3 Persistent challenges in fracture risk prediction

Nine studies addressed fracture risk prediction (31, 53, 68, 78, 79, 82, 84, 85, 89), encompassing heterogeneous endpoints, including binary classification of osteoporotic vertebral compression fractures, discrimination of moderate-to-severe fractures from normal or mild cases, and site-specific outcomes (e.g., lumbar spine, proximal femur). Across these tasks, model performance was both more variable and consistently lower than that observed for bone density-based tasks, with AUCs ranging from 0.63 to 0.99. The lower performance bound, compared with osteoporosis (0.767–1.000) and osteopenia prediction (0.69–1.000), underscores the intrinsic complexity of fracture risk, which depends on multiple factors beyond bone mineral density, including fall propensity and skeletal structure.

The lowest AUC (0.63) was reported in a Finnish study using DXA with prospective validation, representing the only model evaluated in a prospective cohort (84). In contrast, the highest AUC (0.99) was observed in a U. S.-based, multicenter X-ray study restricted to male participants but without external validation. Among studies with external validation (*n* = 4) (53, 68, 84, 89), the AUC ranged from 0.63 to 0.94, with a median of approximately 0.82. In contrast, studies without external validation (*n* = 5) reported a higher median AUC of approximately 0.88 (range: 0.79–0.99). This pattern suggests that the absence of external validation may lead to overestimation of model performance.

Descriptive stratification highlighted the relative scarcity of fracture risk studies (Table 6). CT-based deep learning models (*n* = 1) achieved an AUC of 0.83, while traditional ML/hybrid models on CT showed a median AUC of 0.83 (0.79–0.88). X-ray-based deep learning models (*n* = 3) reported a higher median AUC of 0.94, but with limited external validation.

Across tasks, fracture prediction demonstrated lower median discrimination (AUC = 0.83) compared with osteoporosis (0.93) and osteopenia prediction (0.93), with a broader lower tail. Descriptive stratification by imaging modality and model type (Table 6) further highlights these differences. Deep learning approaches generally achieved high median AUCs in osteoporosis (0.94 for CT) and osteopenia prediction, while fracture risk studies were relatively scarce.

These findings indicate that while imaging-based models perform well for bone density classification, fracture risk prediction remains substantially more challenging. CT-based opportunistic screening currently dominates the literature, with limited evidence available for MRI and DXA modalities. Clinicians should interpret reported performance metrics in the context of the specific imaging modality, predicted outcome, and model type. Future high-quality studies with robust external validation across diverse modalities and fracture-focused endpoints are urgently needed to bridge these gaps.

### 3.6 Sources of heterogeneity: drivers of study-level variation and performance variability

**Figure 3:**
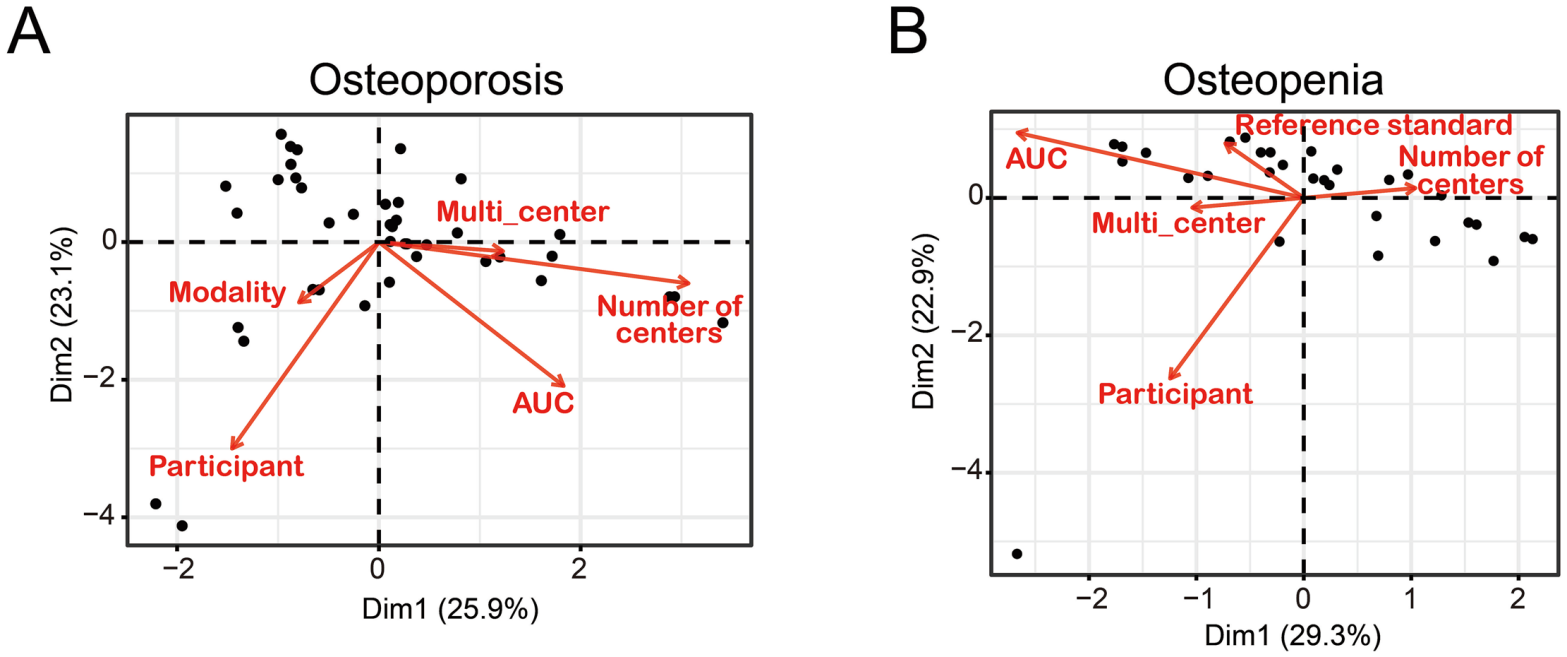
Principal component analysis (PCA) of study-level heterogeneity. **(A)** PCA based on studies predicting osteoporosis. **(B)** PCA based on studies predicting osteopenia. The PCA was conducted using 12 key study-level variables from the included studies. The first two principal components (*PC1* and *PC2*) are displayed. Red arrows represent the loading vectors of individual variables, indicating the direction and magnitude of their contribution to variance in the PCA space. The relative orientation and length of the arrows reflect correlations among variables and their influence on study heterogeneity. For simplicity, only the variables with the largest contributions to *PC1* and *PC2* are shown.

To identify sources of heterogeneity, we performed principal component analysis on 12 variables extracted from 57 studies, including reference standard, AUC, participant number, external validation, multicenter design, number of centers, country, study design, gender, ROI category, modality, and image-based extraction strategy. Because osteoporosis and osteopenia represent distinct prediction tasks, analyses were conducted separately (Figure 3).

In osteoporosis prediction, the first two principal components explained 49% of the variance (PC1: 25.9%; PC2: 23.1%). PC1 was mainly associated with the number of centers (loading = 0.67), AUC (loading = 0.40), and participant number (loading = −0.32), while PC2 was driven by participant number (loading = −0.70) and AUC (loading = 0.40). In osteopenia prediction, the first two components explained 52.2% of the variance (PC1: 29.3%; PC2: 22.9%). PC1 was primarily associated with AUC (loading = 0.71), participant number (loading = −0.33), and multicenter design (loading = −0.28), and PC2 with participant number (loading = −0.79), AUC (loading = 0.29), and reference standard (loading = 0.24).

Across both tasks, smaller studies tended to report higher AUC. This pattern likely reflects multiple converging mechanisms rather than a single cause. Smaller studies are more susceptible to overfitting, as models trained on limited data may capture noise rather than generalizable signal. They may also be affected by selection bias, where the study cohort is more homogeneous than the target population, artificially inflating performance.

Single-center studies generally showed higher performance than multicenter studies, a finding that may be explained by institution-specific acquisition protocols and population characteristics that reduce data variability and make the prediction task appear easier than it would be in a heterogeneous real-world setting. In multicenter studies, the inclusion of diverse scanners, protocols, and patient demographics introduces greater variability that challenges model generalization, resulting in slightly lower but more realistic AUC estimates.

Overall, these results indicate that participant number, multicenter design, and the number of centers is key structural factors that contribute to the observed heterogeneity in reported model performance, and that the higher AUCs in smaller, single-center studies should be interpreted with appropriate caution.

## 4 Discussion

This systematic review synthesizes evidence from 57 studies published between 2019 and 2026, documenting the rapid development of AI-based opportunistic screening for osteoporosis. To our knowledge, this is the first comprehensive synthesis to characterize methodological heterogeneity, modeling trends, and performance variability in this field. The findings provide several insights relevant to clinical translation and future study design.

The marked increase in publications since 2023, with nearly 80% of included studies published in the last 3 years, reflects a rapid expansion of the field. This pattern is consistent with trends in medical imaging AI, where convolutional neural networks have largely replaced traditional machine learning methods across diagnostic tasks (30, 90–92).

### 4.1 Transition from handcrafted radiomics to deep learning architectures

Our analysis shows a clear temporal shift in modeling strategies. Studies from 2019 to 2022 mainly used handcrafted radiomics features or Hounsfield unit measurements with conventional machine learning classifiers. Since 2023, deep learning architectures such as ResNet, DenseNet, and U-Net have become dominant. These approaches enable end-to-end learning, reduce reliance on manual feature engineering, limit interobserver variability in ROI delineation, and capture image patterns beyond predefined radiomic features (93, 94).

In 2025, studies began to fine-tune large pretrained models, including OpenCLIP, DINOv2, and CheXagent, indicating a move toward foundation models. This trend aligns with advances in natural language processing and computer vision, where pretrained models adapted to specific tasks often outperform models trained from scratch (95, 96). For osteoporosis screening, such models may learn robust representations of vertebral structure and trabecular texture, improving performance in small datasets and enhancing generalizability across imaging protocols.

This progression also introduces limitations. Deep learning models, especially those using whole image inputs without predefined ROIs, often lack interpretability. Although several studies reported excellent performance using whole-image approaches (AUCs 0.82–0.99), these models often provide limited insight into the image regions driving predictions. Without explicit vertebral localization, predictions may be influenced by non-causal image characteristics, reducing model transparency. In contrast, radiomics-based models, despite slightly lower performance, retain greater feature-level interpretability and facilitate biological and clinical interpretation.

### 4.2 Predictive value of clinical features

Several high-quality studies showed that imaging-only models achieved performance comparable to or better than models that combine imaging and clinical variables, suggesting that deep learning models may implicitly infer age, sex, and certain laboratory derangements directly from images. However, such findings do not imply that clinical features are redundant. A recent study demonstrated that a model relying exclusively on 35 routine clinical variables—without any imaging input—achieved 83.94% accuracy for osteoporosis screening across multiple hospitals (87). This illustrates that clinical features carry substantial independent discriminative power. The modest gains observed in earlier multimodal models may therefore reflect ceiling effects when imaging already captures the relevant physiological information, rather than an inherent limitation of clinical data. Future work should systematically explore both standalone clinical models and fusion architectures, and define the specific contexts—such as suboptimal image quality, limited scanner access, or atypical disease presentations—where clinical variables offer the greatest added value.

### 4.3 Performance heterogeneity and task complexity

Model performance varied widely across studies, with AUC for osteoporosis prediction ranging from 0.767 to 1.000. This variability reflects both differences in task complexity and methodological factors. Principal component analysis identified participant number and multicenter design as key drivers, with smaller single-center studies consistently reporting higher performance. This pattern indicates overfitting and optimism bias in studies with limited sample size and data diversity.

Reports of near-perfect performance require caution. Eight studies achieved AUC ≥ 0.98, including four with an AUC of 1.000. Perfect classification in medical diagnosis is exceptionally rare, given inherent biological variability and measurement error in reference standards (97). These findings likely reflect overfitting, spectrum effects, or limited case heterogeneity rather than true diagnostic perfection.

Performance also differed by task. Bone density classification, such as osteoporosis diagnosis based on a T score threshold, provides a relatively consistent reference. In contrast, fracture risk prediction is inherently probabilistic and influenced by multiple factors beyond bone mineral density, including biomechanics and tissue properties (98, 99). This complexity is reflected in lower and more variable performance, with AUC values as low as 0.63.

Notably, the only prospectively evaluated model for fracture prediction reported the lowest performance, suggesting that prospective studies yield more realistic estimates than retrospective analyses.

### 4.4 Validation gap as a barrier to clinical translation

Although PROBAST considers absence of external validation a high risk of bias, only 20 studies (36%) included any external validation, and independent multi-institutional evaluation was rare. The single international validation study illustrates both feasibility and scarcity.

This gap directly affects reported performance. Models tested on data from the same institution, scanner, and population tend to overestimate performance compared with evaluations in new settings. Beyond the absence of external validation itself, scanner- and protocol-related heterogeneity constitutes an additional threat to model generalizability. Attenuation values can vary substantially across tube voltages and scanner models, undermining the portability of threshold-based or attenuation-based screening tools. A recent landmark study directly tackled this issue by developing a statistical correction method to normalize trabecular attenuation across 43 scanner models and six tube voltages, using a dataset of over 538,000 CT examinations (100). The automated ROI placement achieved greater than 99% agreement with manual review, and the derived normative values and diagnostic thresholds demonstrated consistent performance across diverse populations, establishing a reproducible framework for multi-scanner opportunistic screening. This study illustrates that rigorous, large-scale validation with explicit scanner calibration is not only possible but essential for clinical translation.

Nonetheless, such comprehensive efforts remain the exception. Model development requires substantial resources for data curation, annotation, and training, while data sharing is constrained by regulatory and technical barriers. Without the level of validation and standardization, the clinical utility of opportunistic screening tools will remain uncertain. Future research should therefore prioritize multi-center, multi-scanner designs with built-in calibration strategies to bridge the gap between promising research and real-world deployment.

### 4.5 Limitations of this review

This review has several limitations. First, although a pre-specified hierarchical extraction strategy was applied to select a single AUC from each of the 57 included studies, this approach may still have introduced a degree of optimistic bias. Specifically, the final last-resort criterion, “selecting the highest reported AUC,” was required in only 8 studies (14.0%). In most studies, the AUC explicitly reported in the abstract was extracted, while external validation results were consistently prioritized over internal validation whenever both were available. Among the 8 studies requiring the last-resort criterion, Study ID 7 reported multiple external validation cohorts without identifying a preferred validation result; Studies 9 and 13 performed external validation but did not clearly distinguish the external validation cohort in the reported results; Study 55 compared clinical, radiomics, and combined clinical–radiomics models without specifying a primary model; Study 33 reported two independent test cohorts without indicating which should be considered the primary validation cohort; and only 3 studies required direct selection of the numerically highest reported AUC without additional contextual guidance. Therefore, although selecting the highest AUC may have introduced some optimistic bias, this occurred in only a small minority of included studies and largely reflected inconsistent reporting rather than arbitrary selection. Because this review aimed to provide a descriptive overview rather than a quantitative meta-analysis of diagnostic performance, we believe that the potential influence of this approach on our overall conclusions is likely to be modest. Nevertheless, the extracted AUC values should be interpreted cautiously, as they may represent upper-bound estimates of model performance rather than the performance typically expected in routine clinical practice.

Second, study-level heterogeneity precluded formal meta-analysis. Therefore, we used PCA to illustrate the sources of heterogeneity across studies. Third, inconsistent reporting across studies, particularly for model calibration, clinical utility, and exclusion criteria, limited the assessment of model quality. Fourth, the focus on model development rather than validation studies means that reported performance likely reflects upper bounds rather than real-world effectiveness. Fifth, although our search strategy (see Supplementary Table 2) combined multiple keywords (including “opportunistic,” “incidental,” “unsuspected,” and “asymptomatic”) to balance sensitivity and specificity, some functionally opportunistic studies may still have been missed. For instance, a relevant MRI study (86) that came to our attention during the review process was not captured by the initial search, indicating that certain articles using routine imaging for bone density assessment without these specific terms in their titles or abstracts may have been inadvertently excluded. Sixth, only English-language publications were included, which may have introduced both language and publication bias. Given the rapid expansion of AI research in medical imaging across non-English-speaking countries, relevant studies published in local-language journals may have been missed. This restriction may reduce the global representativeness of the available evidence and underrepresent variations in imaging protocols, healthcare settings, and patient populations. Furthermore, studies reporting favorable model performance are more likely to be published in international English-language journals, potentially leading to an overly optimistic assessment of the current diagnostic performance and clinical readiness of AI-based opportunistic osteoporosis screening. This residual gap should be considered when interpreting the scope of our findings.

## 5 Conclusion

This review highlights the rapid growth of AI-based opportunistic screening for osteoporosis, marked by a shift toward deep learning and more advanced modeling approaches. However, progress in model development has not been matched by advances in validation. The dominance of retrospective, single-center studies and the lack of external validation remain key barriers to clinical translation. Although current evidence supports technical feasibility, the next phase requires multi-institutional collaboration, prospective evaluation, and standardized reporting. These steps are essential to translate current models into reliable clinical tools for osteoporosis detection and fracture prevention.

## Data Availability

The original contributions presented in the study are included in the article/Supplementary material, further inquiries can be directed to the corresponding author.
